# Enhancing the Catalytic Performance of Candida antarctica Lipase B by Chemical Modification With Alkylated Betaine Ionic Liquids

**DOI:** 10.3389/fbioe.2022.850890

**Published:** 2022-02-21

**Authors:** Yu Xue, Xiao-Guang Zhang, Ze-Ping Lu, Chao Xu, Hua-Jin Xu, Yi Hu

**Affiliations:** School of Pharmaceutical Sciences, State Key Laboratory of Materials-Oriented Chemical Engineering, Nanjing Tech University, Nanjing, China

**Keywords:** lipase, ionic Liquid, chemical modification, molecular alteration, molecular simulation

## Abstract

Various betaine ionic liquids composed of different chain lengths and different anions were designed and synthesized to modify *Candida antarctica* lipase B (CALB). The results showed that the catalytic activity of all modified lipases improved under different temperature and pH conditions, while also exhibiting enhanced thermostability and tolerance to organic solvents. With an increase in ionic liquid chain length, the modification effect was greater. Overall, CALB modified by [BetaineC_16_][H_2_PO_4_] performed best, with the modified CALB enzyme activity increased 3-fold, thermal stability increased 1.5-fold when stored at 70°C for 30 min, with tolerance increased 2.9-fold in 50% DMSO and 2.3-fold in 30% mercaptoethanol. Fluorescence and circular dichroism (CD) spectroscopic analysis showed that the introduction of an ionic liquid caused changes in the microenvironment surrounding some fluorescent groups and the secondary structure of the CALB enzyme protein. In order to establish the enzyme activity and stability change mechanisms of the modified CALB, the structures of CALB modified with [BetaineC_4_][Cl] and [BetaineC_16_][Cl] were constructed, while the reaction mechanisms were studied by molecular dynamics simulations. Results showed that the root mean square deviation (RMSD) and total energy of modified CALB were less than those of native CALB, indicating that modified CALB has a more stable structure. Root mean square fluctuation (RMSF) calculations showed that the rigidity of modified CALB was enhanced. Solvent accessibility area (SASA) calculations exhibited that both the hydrophilicity and hydrophobicity of the modified enzyme-proteins were improved. The increase in radial distribution function (RDF) of water molecules confirmed that the number of water molecules around the active sites also increased. Therefore, modified CALB has enhanced structural stability and higher hydrolytic activity.

## 1 Introduction

Lipase (EC 3.1.1.3) has excellent catalytic characteristics such as high selectivity, high stability, requiring mild reaction conditions and the ability to function without a coenzyme. It can efficiently catalyse various reactions, such as hydrolysis, esterification, ammonolysis and the formation of carbon-carbon bonds. Lipase is widely used in various industrial applications, including pharmaceutical, chemical, food, and energy production (Hudlicky, 2011; Reetz, 2013; Shoda et al., 2016). However, lipase can easily be deactivated in non-natural environments, organic solvents or high temperature and extreme pH conditions, significantly limiting the potential for large-scale industrial applications. Therefore, molecular modification of lipase to enhance its catalytic performance is of great practical value, providing novel modified enzyme varieties with enhanced activity, stability, specificity and environmental tolerance for industrial application, allowing it to adapt to the specific requirements of industrial production processes (Strohmeier et al., 2011; Bornscheuer et al., 2012).

Chemical modification is a commonly used method for the molecular modification of enzymes. Previous research has shown that chemical modification of lipases is an effective and valuable strategy, having the advantages of low costs and laboratory feasibility, allowing new enzymatic properties to be rapidly and easily created (Diaz-Rodriguez and Davis, 2011; Rodrigues et al., 2011; Zhang et al., 2015). However, the types of modifiers commonly used are limited and it has proven difficult to significantly improve the enzymatic properties of lipase even with modification using multiple modifiers. For example, *Candida rugosa* lipase (CRL) was successfully modified by citraconic anhydride, although the effect of modification on the activity and thermostability of lipase was negligible and further modification of lysine residues caused a reduction in thermostability (Matsumoto et al., 2016). Therefore, in order to meet the required level of modification diversity, new modifiers must be identified. In previous studies, functional ionic liquids (ILs) have been used as modifiers to enhance the catalytic performances of various lipases simultaneously, with results showing that different combinations of cations and anions in ILs impact the catalytic performance of modified lipases. For example, the thermal stability of *Candida antartica* lipase B(CALB) modified with imidazole functional ionic liquid [HOOCBMIM][Cl] was increased 7-fold at 70°C and 5-fold in methanol (50% v/v), with a slightly higher hydrolytic activity than unmodified CALB (Jia et al., 2013a). Amino acid ionic liquids (AAILs) with a chiral structure were used to modify CALB, resulting in improved catalytic activity under different temperature and pH conditions, with enhanced thermostability and tolerance to organic solvents (Xu et al., 2021). In a previous study, imidazole, choline and proline ionic liquids were designed and synthesized to chemically modify CRL and PPL, with results showed that the enzyme activity, thermal stability, organic solvent tolerance and other enzymatic properties of the modified enzymes were improved (Jia et al., 2013b; Li et al., 2015; Xu et al., 2019). Overall, these findings show that ionic liquids are highly effective chemical modifiers.

While the effectiveness of ionic liquids for enhancing the stability and activity of proteins has been validated, the corresponding molecular mechanisms remain poorly understood. Recent studies have shown that molecular simulation allows the change in enzymatic properties and reaction mechanism to be reasonably predicted, providing helpful guidance for the modification of enzymes at the molecular level (Frushicheva and Warshel, 2012; Chen et al., 2013; Qu et al., 2018). In addition, molecular simulation has previously been applied to explain the mechanism of change in the catalytic performance of lipases modified using ionic liquids, exhibiting great potential in enzyme engineering applications. For example, molecular dynamics (MD) simulation has been successfully used to establish the mechanisms of improvement in the structural stability and catalytic efficiency of porcine pancreas lipase modified with [HOOCBMIM] [Cl] and CALB modified with [N-AC-L-Pro] [Cl] (Zhang et al., 2016; Xu et al., 2021).

According to previously reported literature, betaine ionic liquid can be used as reaction medium to participate in a series of chemical reactions, with a diverse range of uses including medical, cosmetic and chemical industry applications (Zhu et al., 2017; Parajó et al., 2019). In addition to extracting betaine compounds from natural products, alkylated betaine ionic liquids can also be obtained by quaternization of aliphatic tertiary amines, allowing the design and synthesis of betaine ionic liquids with different chain lengths and anions.

CALB is one of the most widely studied lipases, differing from other lipases as it has no lid structure and does not exhibit interfacial activation, while the active centre is exposed allowing the substrate uncontrolled access. As a result, CALB is sensitive to high temperatures, solvents and extreme pH environments, significantly reducing enzyme activity and further limiting the potential for industrial application of CALB. In this study, CALB was chemically modified using an alkylated betaine ionic liquid, with analysis of the degree of modification and the resulting activity, organic solvent tolerance and thermal stability. Both fluorescence spectroscopy and circular dichroism (CD) spectroscopy were carried out to investigate the changes in enzyme structure and establish the catalytic properties associated with the changes. MD simulation was performed to identify the mechanism behind the enhanced structural stability and activity of modified CALB. The aim of this study was to develop a novel, efficient and practical method to enhance the catalytic performance of lipases lacking a lid domain and establish the molecular mechanisms of enhanced catalytic activity. A complete and coherent method to study lipase modification is proposed, which provides rational guidance for the design of functional ionic liquids.

## 2 Methods and Materials

### 2.1 Materials

Lipozyme CALB (10 mg/ml) was purchased from J&K Chemicals Co. Ltd. p-Nitrophenyl palmitate (pNPP, 98%), p-nitrophenol (99.5%), dithiothreitol, and 2,4,6-trinitrobenzenesulfonic acid solution (5% w/v in H_2_O) were purchased from Sigma-Aldrich (China). The bicinchoninic acid (BCA) protein assay kit was obtained from Nanjing Juyou Instrument Co. Ltd. Carbonyldiimidazole (CDI, 97%), iodoacetamide, and trifluoroacetic acid were obtained from Aladdin Chemistry Co. Ltd. Trypsin was purchased from Promega (Beijing) biotech Co. Ltd. All reagents were of analytical grade or higher and were used without any further purification.

### 2.2 Synthesis of Modifiers

**FIGURE 1:**
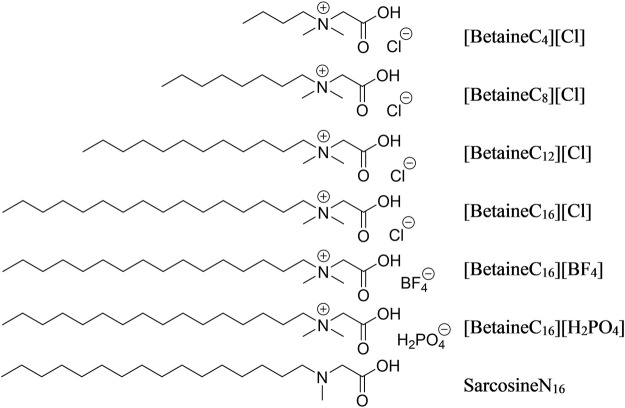
Structure of the seven modifiers.

The synthesis of ionic liquids was referred to previous reports (Li et al., 2012; Kharlamov et al., 2014; Tcarkova et al., 2015). 0.01 mol of chlorobutane (chlorooctane, chlorododecane, chlorohexadecane) was added to a 33% (v/v) dimethylamine aqueous solution using a constant pressure drop funnel and the reaction was maintained for 1 h in an ice bath, then heated under reflux at 50–100°C for 24 h. The reaction solution was then cooled to room temperature and washed three times with distilled water to obtain a yellow oily liquid. Chloroacetic acid and sodium hydroxide were mixed and stirred at a molar ratio of 1:1.2 for 10 min, then the mixture was added to the yellow oily liquid and heated under reflux at 95°C for 4 h. The reaction solution was disposed with an excess of concentrated hydrochloric acid, passed through a filter and the white powder residue was collected. The powder was recrystallized in ethanol and ether to form a hexadecyl betaine chloride ionic liquid. The structures of all modifiers are shown in Figure 1.

The hexadecyl betaine chloride ionic liquid was added to an acetone solution, with excess sodium dihydrogen phosphate and sodium fluoroborate added sequentially. The reaction solution was stirred at room temperature for 48 h. The reaction solution was then filtered to remove the precipitate and the acetone solution was distilled to generate a hexadecyl betaine ionic liquid containing H_2_PO_4_ and BF_4,_ In order to assess the suitability of the ionic liquid as a modifier, hexadecyl sarcosine was synthesized by dissolving sarcosine in methanol, then slow drop plus two sulfoxide chloride, methyl oily creatine. Then, methyl sarcosine and chlorinated cetane were added to methanol along with a small amount of triethylamine as an acid binding agent and the mixture was heated under reflux for 12 h. The obtained solution as dried to remove the methanol, then extracted using ethyl acetate and the organic phase was dried, forming the crude product and then recrystallized using a mixture of acetone and petroleum ether to form the pure product cetylcreatine. A detailed overview of the characterization of modifiers is shown in the Supplementary Material.

### 2.3 Purification of Lipase

The crude Lipozyme CALB enzyme solution (25 ml) was centrifuged at 4°C for 30 min, with the supernatant then collected and dialyzed through a 10 kDa dialysis membrane against ice-cold water for 16 h to remove excess salts. After dialysis, the purified enzyme preparation was stored in the dark at 4°C.

### 2.4 Chemical Modification and Determination of Modification Degree

The synthesized betaine ionic liquid (1 mmol) was combined with carbonyl diimidazole (CDI) (1 mmol) and then 2 ml anhydrous dimethyl sulfoxide was added as the reaction solvent. The reaction was maintained with continual agitation via a magnetic stirrer (300 rpm), at room temperature for 2 h. The activated modifier was then added to the enzyme solution and the reaction continued in an ice bath with continual agitation using a magnetic stirrer (300 rpm) for 8 h. At the end of the reaction period, the modified enzyme reaction solution was transferred directly into a 10 KDa dialysis bag and maintained for 24 h in an ice bath, with dialysis repeated every 6 h. In order to estimate the number of modified lysine residues, trinitrobenzene sulfonic acid method was used to determine the degree of modification (Snydr and Sobocinski, 1975).The seven modified enzymes were finally obtained, with (1) the enzyme modified using [BetaineC_4_][Cl] named [BetaineC_4_][Cl]-CALB; (2) the enzyme modified using [BetaineC_8_][Cl] named [BetaineC_8_][Cl]-CALB; (3) the enzyme modified using [BetaineC_12_][Cl] named [BetaineC_12_][Cl]-CALB; (4) the enzyme modified using [BetaineC_16_][Cl] named [BetaineC_16_][Cl]-CALB; (5) the enzyme modified using [BetaineC_16_][BF_4_] named [BetaineC_16_][BF_4_]-CALB; (6) the enzyme modified using [BetaineC_16_][H_2_PO_4_] named [BetaineC_16_][H_2_PO_4_]-CALB; (7) the enzyme modified using Sarcosine named SarcosineN_16_-CALB.

### 2.5 Determination of Enzyme Activity

The activity of native and modified CALBs were measured according to the hydrolysis of pNPP to pNP (p-nitrophenol). The pNPP solution (16.5 mM) was mixed with phosphate buffer (50 mM) containing 0.4% (w/v) Triton X-100 and 0.1% (w/v) Arabic gum (1:9 v/v). The reaction was initiated by the addition of 10 μl lipase solution and 240 μl substrate solution (diluted to an appropriate concentration). The pNP reaction product was monitored by measuring the absorbance at 405 nm using a microplate reader (SpectraMax190, Molecular Devices, LLC, United States), with one unit of lipase activity defined as the amount of lipase releasing 1 μmol of pNP from pNPP per minute. All experiments were performed in triplicate.

### 2.6 Determination of Enzyme Activity Under Different Temperature and pH Conditions

At a constant temperature of 37°C, the effect of varying pH (phosphate buffer pH 6.0, 6.5, 7.0, 7.5, 8.0) on the activity of native and modified CALBs was measured using the pNPP hydrolysis assay, as described above. The effect of varying temperature on the activity of native and modified CALBs was measured under optimum pH conditions, with temperatures ranging from 15 to 70°C, at increasing intervals of 5°C. All experiments were performed in triplicate.

### 2.7 Determination of Thermal Stability

The native and modified CALB enzymes were maintained at 70C, with 10 μl samples collected every 10 min to measure the enzyme activity under optimum pH and temperature conditions using the pNPP hydrolysis assay as described above.

### 2.8 Determination of Tolerance to Organic Solvents

The native and modified CALB enzymes were stored in varying concentrations of dimethyl sulfoxide (0–50%) for 2 h at room temperature, with the enzyme activities tested after 2 h under optimum conditions using the pNPP hydrolysis assay as described above.

### 2.9 Determination of Tolerance of Denaturing Agents

The native and modified CALB enzymes were stored in varying concentrations of mercaptoethanol (0–30%) for 2 h at room temperature, with the enzyme activities tested after 2 h under optimum conditions using the pNPP hydrolysis assay as described above.

### 2.10 Determination of Enantiomer Selectivity

**FIGURE 2:**
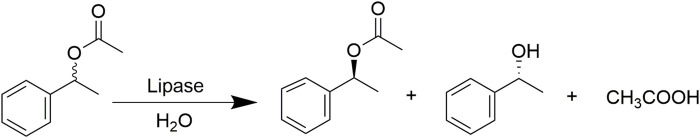
Lipase catalysed hydrolysis of (R,S) -1-phenylethyl acetate.

As shown in Figure 2, the enantio-selectivity of native and modified CALB enzymes was determined by adding (R,S) -1-phenyl acetate (30 mM) into a 5 ml buffer solution (0.25 M, pH 7.5). After magnetic stirring for 10 min, 5 mg of lyophilized enzyme powder was added and the reaction was carried out under the previously established optimal temperature and pH conditions. At specified intervals, 0.2 ml samples were collected and extracted with ethyl acetate, with the organic phase dried with sodium sulfate and the selection rate was determined by high performance liquid chromatography. Detection was performed using a Chiralcel Oz-H chiral column and a UV detector (254 nm) with N-hexane and isopropanol mobile phases (v/v: 95/5) at a flow rate of 0.3 ml/min and a column temperature of 25°C. The reaction calculations are shown in Eqs 1–4 as follows:

$$ee_{S}=\frac{S-R}{R+S}\times 100\text{\%}$$ (1)

$$ee_{P}=\frac{P-Q}{P+Q}\times 100\text{\%}$$ (2)

$$∁\;=\frac{ee_{S}}{ee_{P}+ee_{S}}\times 100\text{\%}$$ (3)

$$\text{E}=\ln\left[1-∁\left(1+ee_{P}\right)\right]/\ln\left[1-∁\left(1+ee_{P}\right)\right]$$ (4)

### 2.11 Determination of Fluorescence Spectra

At room temperature, the original enzyme and the modified enzyme solutions were diluted to the same concentration with distilled water and 2 ml of the enzyme solution was then placed in a colorimetric dish for determination by fluorescence spectrophotometry at an excitation wavelength of 295 nm and a wavelength scanning range from 280 to 400 nm.

### 2.12 Determination of Circular Dichroism (CD)

At room temperature, the original enzyme and the modified enzyme solutions were diluted to the same concentration with distilled water and 2 ml was removed and placed in a colorimetric dish. A circular dichrometer was used to determine the baseline at a scanning speed of 50 nm/min and a scanning wavelength range of 190–260 nm. The obtained data was processed using CDPro software, and the secondary structure content change was calculated.

### 2.13 MALDI-TOF-MS

Chemical modification reactions are typically non-specific in nature, resulting in difficulty directly identifying modified sites. Matrix-assisted laser desorption/ionization time-of-flight mass spectrometry (MALDI-TOF-MS) is a powerful tool which can be used to solve the problem. [BetaineC_4_][Cl]-CALB and [BetaineC_16_][Cl]-CALB were digested using a previously reported method (Xu et al., 2021), with CALB then isolated, purified and detected by SDS-PAGE gel electrophoresis. The purified samples were freeze-dried and the resulting CALB enzyme powder was dissolved in 0.1% trifluoroacetic acid. α-cyano-4-hydroxy cinnamic acid (HCCA) was used as the matrix for MALDI-TOF-MS detection.

### 2.14 Molecular Dynamics Simulation

The modification results in this study showed that CALB modified with [BetaineC_4_][Cl] exhibited the lowest hydrolytic activity and stability, while CALB modified with [BetaineC_16_][Cl] exhibited the highest hydrolytic activity and stability. Therefore, these two modified enzymes were used for the theoretical calculations discussed below. The modified CALB model was obtained using Discovery studio 2016 software to mutate CALB modification sites into non-natural amino acid residues. Gromacs software (version 2018.1, Gromacs54a7 force field) was used to simulate CALB lipase dynamics before and after modification. The temperatures were set at room temperature (300 K) and the optimum temperatures of native and modified CALB. The optimal configuration was obtained by minimizing the charge balance energy of the structural model, with the positional restriction simulation of temperature and pressure carried out using the coupled V-Resale algorithm and Parrinello-Rahman algorithm, respectively. After positional restriction simulation, the system with positional equilibrium was simulated for 20 ns. Lincs algorithm was adopted with a system step size of 0.002 ps. Protein structure and energy information were collected every 1 ps. Detailed information of the processing of initial structures and construction of force fields of non-natural amino acid residues are provided in the Supplementary Material.

## 3 Results

### 3.1 Modification Degree and Optimal Enzyme Activity

As shown in Table 1, the degree of modification of CALB by alkylated betaine ionic liquids with varying chain lengths did not differ significantly. This result is in contrast to previously reported results, in which the degree of modification of CALB modified by imidazole-type and chiral proline-type ionic liquids were significantly different (Jia et al., 2013a; Xu et al., 2019). In the present study, with increasing chain length the enzyme activity increased accordingly. The enzyme activity of [BetaineC_16_][Cl]-CALB increased by 2.75-fold, while after anion exchange the activity of [BetaineC_16_][H_2_PO_4_]-CALB increased by 3.06-fold. It is of note, that the level of activity promotion observed in the present study was higher than previously reported for chiral proline modified CALB (Xu et al., 2021). Moreover, results show that the modification effect of cetylsarcosine on CALB was significantly lower than the effect of betaine ionic liquids, including butylbetaine chloride ionic liquid, which further proves the superiority of ionic liquids as modifiers.

**TABLE 1:** Modification degree and optimal enzyme activity of the native and modified CALBs.

Enzyme	Degree of modification (%)	Hydrolytic activity (U/g)	Relative activity (%)
CALB		263.84	100
[BetaineC_4_][Cl]-CALB	42.24 ± 1.34	485.76 ± 6.54	184.11
[BetaineC_8_][Cl]-CALB	41.39 ± 1.13	540.13 ± 5.45	204.71
[BetaineC_12_][Cl]-CALB	40.92 ± 1.27	580.67 ± 7.23	220.08
[BetaineC_16_][Cl]-CALB	40.81 ± 1.14	724.56 ± 6.52	274.62
[BetaineC_16_][BF_4_]-CALB	39.74 ± 1.08	603.65 ± 5.96	228.79
[BetaineC_16_][H_2_PO_4_]-CALB	42.56 ± 1.22	806.74 ± 6.64	305.76
SarcosineN_16_-CALB	40.87 ± 1.19	270.42 ± 6.87	102.49

### 3.2 Enzymatic Activity of Native and Modified Enzymes at Different Temperatures and pH

**FIGURE 3:**
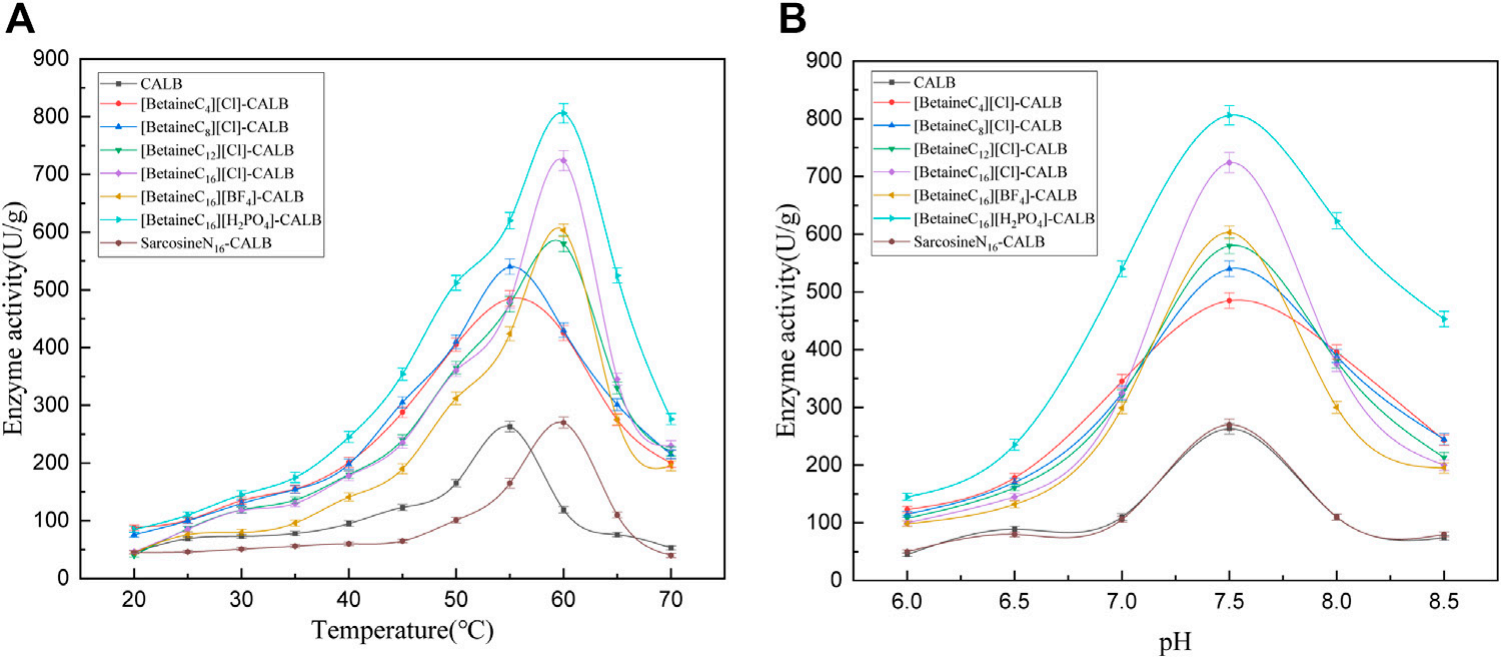
Enzymatic activity of CALBs at different reaction temperatures **(A)** and pH **(B)**.

As shown in Figure 3A, the activity of the modified enzymes improved to varying degrees, with the optimal reaction temperature of enzymes changing after modification. With an increase in chain length, the optimal temperature of modified enzymes changed from 55 to 60°C. At lower reaction temperatures, the activity of modified enzymes were only slightly increased compared with that of the original enzyme. At temperatures of 35°C and above, the activity of modified enzymes significantly improved, with the highest increase in activity observed for [BetaineC_16_][H_2_PO_4_]-CALB. When the temperature was increased to higher than the optimum temperature, the modified enzymes exhibited improved tolerance and maintained a high enzyme activity at temperatures up to 70°C. This may be due to the hydrophobic structure of long chain alkyl betaine ionic liquids, resulting in a relatively hydrophobic microenvironment for the modified enzymes and avoiding direct exposure to high temperatures and solvents, while proteins and long chain alkanes can effectively enhance the rigid structure of the enzyme (Wan et al., 2017; Dahanayake et al., 2019).As shown in Figure 3B, the optimal pH for both the native and modified enzyme was 7.5, resulting in the activity of all modified enzymes being improved. Consistent with the observed effect of temperature on enzyme activity, the [BetaineC_16_][H_2_PO_4_]-CALB modified enzyme exhibited the highest enzyme activity, with a relatively stable performance at pH < 6.5, while activity was significantly improved compared with the original enzyme at pH > 6.5. When the optimal pH of 7.5 was exceeded, the modified enzymes retained higher activities compared to the native enzyme, with the activity of [Betainec_16_][H_2_PO_4_]-CALB most obviously increased, indicating that this modified enzyme could adapt to a wider pH range than the native enzyme.

### 3.3 Thermostability of Native and Modified Enzymes

**FIGURE 4:**
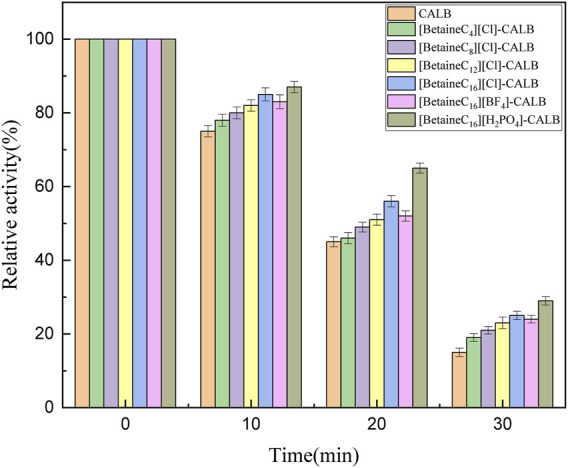
Thermal stability of native and modified CALBs at 70°C.

Thermal stability is an important enzymatic property. As shown in Figure 4, the thermal stability of the native and modified enzymes were significantly changed after 30 min of being maintained at 70°C, with the activity of the native enzyme almost completely inhibited, while the modified enzymes remained active, indicating that modification improved the thermal stability of lipase. With an increase in chain length, a more hydrophobic environment was observed on the surface of the modified enzymes. Covalent attachment of the modifier onto the protein surface results in the conformation of the modified enzyme being more rigid, allowing the active conformation of the modified enzyme to avoid damaged by high temperatures. It has previously been reported that different anions in the system also influence the thermal stability of the enzyme (Grochulski et al., 1993; Yang, 2009). It has previously been reported that when the ionic liquid contains K (Kosmotropic)-type cations, the anion is closer to C (Chaotropic)-type, resulting in the modified enzyme having better thermal stability (Jia et al., 2013a). However, in the present study the thermal stability of [BetaineC_16_][H_2_PO_4_]-CALB modified enzyme was highest, which is inconsistent with the results of previous studies, as the cation is a C-type ion and the anion is closer to K-type, resulting in better stability in the modified enzyme. While more research is required to fully understand the role of ion types in thermal stability, these results lay a foundation for the design of ionic liquid modifiers with different cation and anion combinations for improved lipase thermal stability.

### 3.4 Organic Solvent and Denaturing Agents Tolerance of Native and Modified Enzymes

**FIGURE 5:**
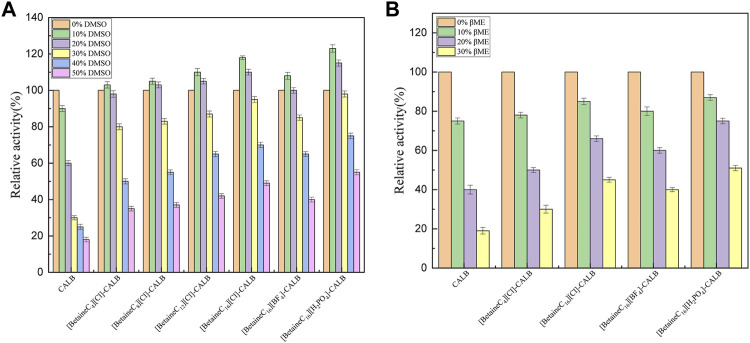
Effect of dimethyl sulfoxide **(A)** and mercaptoethanol **(B)** concentrati on enzyme activities.

Lipase is widely used in the chemical industry. However, the reaction system often involves the use of strong polar solvents, resulting in lipase being easily deactivated and therefore, modification can improve the resistance of lipases to organic solvents and significantly broaden their application range. As shown in Figure 5A, the enzyme activity of the native enzyme decreased with an increase in the volume fraction of DMSO, with the enzyme activity decreasing to 18% of the initial enzyme activity when the DMSO volume fraction reached 50%. In contrast, all the modified enzymes exhibited good DMSO tolerance, with a DMSO volume fraction of 10% resulting in a maximum increase in enzyme activity. [BetaineC_16_][H_2_PO_4_]-CALB retained 55% of its initial enzyme activity in 50% DMSO, which differs from the response previously reported for *Burkholderia cepacia* lipase (BCL) modified using imidazolium choline ionic liquid, in which rapid deactivation of the modified enzyme was observed in 30% DMSO, further demonstrated the superiority of long chain alkylated betaine ionic liquids as lipase modifiers (Xu et al., 2018). As shown in Figure 5B, the tolerance of native and modified enzymes to organic solvents were also assessed using different concentrations of mercaptoethanol (0–30% v/v). The enzyme activity of the native enzyme was significantly reduced when the volume fraction of mercaptoethanol was more than 10%, with the enzyme activity reduced to less than 20% of the initial level at 30% mercaptoethanol. Compared with the native enzyme, the tolerance of [BetaineC_16_][Cl]-CALB and [BetaineC4][Cl]-CALB to mercaptoethanol improved marginally, indicating that longer modifier chain lengths resulted in higher modified enzyme tolerance to mercaptoethanol. After anion exchange, [BetaineC_16_][H_2_PO_4_]-CALB was more tolerant to the organic solvent, maintaining 50% of its initial enzyme activity in 30% mercaptoethanol. [Betainec_16_][H_2_PO_4_]-CALB may exhibit higher charge density and stronger hydration ability as it contains more K-type anions, making it easier for the modified enzyme to form an active hydration layer conformation, providing protecting and preventing mercaptoethanol from destroying the structure of the enzyme protein (Zhao et al., 2006).

### 3.5 Enantio-Selectivity of Native and Modified Enzymes

As shown in Table 2, the total conversion rate of all modified enzymes improved, with an increase in the modifier chain length causing the total conversion rate to gradually increase. It is of note, that the conversion rate was almost consistent with the optimal enzyme activity performance. The selectivity of [BetaineC_4_][Cl]-CALB and [BetaineC_8_][Cl]-CALB decreased slightly, although with continued increases in chain length, the E value increased compared with that of the native enzyme. Results show that the ionic liquid cetylbetaine induced better selectivity than short-chain alkylated betaine ionic liquids. Longer chain lengths and stronger hydrophobicity of the ionic liquid results in more obvious conformational changes in the modified lipase, forming an active conformation which changes the substrate channel of the modified enzyme, leading to changes in enantioselectivity (Ueji et al., 2003; Mateo et al., 2007).

**TABLE 2:** Native and modified lipase-catalyzed hydrolysis of (R,S)-1-Phenethyl acetate.

Enzyme	C (%)	*ee_S_* (%)	*ee_p_* (%)	E
CALB	43.9	76	97	150.95
[BetaineC_4_][Cl]-CALB	44.2	76	96	112.80
[BetaineC_8_][Cl]-CALB	44.5	77	96	114.55
[BetaineC_12_][Cl]-CALB	46.7	85	97	179.0
[BetaineC_16_][Cl]-CALB	47.0	87	98	282.48
[BetaineC_16_][BF_4_]-CALB	46.7	86	98	275.55
[BetaineC_16_][H_2_PO_4_]-CALB	47.5	89	98	295.61

### 3.6 Fluorescence Spectroscopy Analysis

**FIGURE 6:**
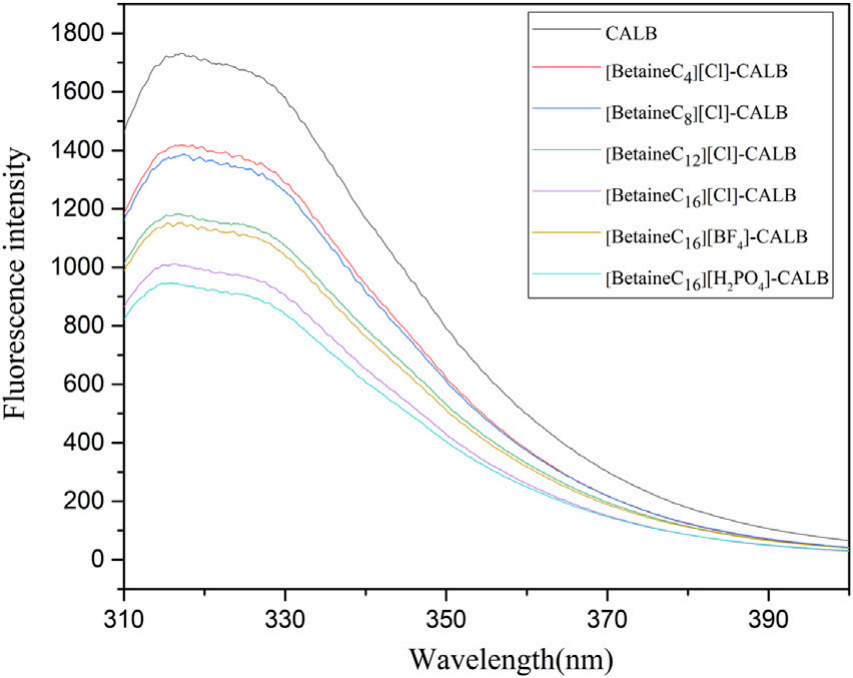
Fluorescence spectra of native and modified enzymes.

The amino acid sequence of lipases often contain fluorescent groups (Trp, Tyr, Phe). When an ionic liquid is bound to the surface of lipase, it causes rapid changes in the microenvironment surrounding lipase fluorescent groups, leading to changes in its maximum absorption wavelength and maximum light absorption intensity (Naka et al., 1997). Fluorescence spectra for the native and modified enzymes are presented in Figure 6, showing that compared to the fluorescence intensity of the native enzyme, modification significantly reduced the fluorescence. As the modifier chain length increased, the fluorescence intensity decreased gradually, with the introduction of the modifier changing the microenvironment surrounding fluorescent residues. Overall, the change in fluorescence intensity was consistent with the change in enzyme activity and the related enzymological properties. The change in modified enzyme fluorescence intensity observed in the present study, was consistent with previously reported results, in which the modification of PPL with imidazole ionic liquids, resulted in the fluorescence spectral intensity decreasing by 7.5–18.2% compared to the native enzyme, indicating that the introduction of ionic liquids increases the exposure of fluoro-groups to hydrophobic environments. Moreover, the maximum absorption wavelength of CALB modified using imidazole-type ionic liquids exhibited a slight blue shift (Jia et al., 2013a), although this effect was not observed in the present study, as the introduction of betaine ionic liquids did not change the maximum absorption wavelength in the modified enzyme fluorescence spectrum.

### 3.7 Circular Dichroism Spectroscopic Analysis

CD spectroscopy can effectively determine the protein secondary structure content, allowing analysis of the relationship between conformational changes in enzyme proteins and activity stability (Mei et al., 2003). The secondary structure of CALB is mainly composed of α/β folds and the correlation between structural and functional changes can be preliminarily analysed by measuring changes in the content of lipase secondary structures before and after modification. According to the reported literature, the decrease in α-helix content is related to an increase in enzyme activity, while the increase in total β-sheet content is associated with increased enzyme stability (Jia et al., 2013b). As shown in Table 3, with increasing carbon chain length, the α-helix content exhibited a decreasing trend, while the total β-sheet content had an increasing trend. The previously discussed enzymological performance experiments confirmed that with an increase in modifier chain length, the modified enzyme exhibited higher activity and better stability, which was consistent with the previously reported literature. In addition, [BetaineC_16_][H_2_PO_4_]-CALB was found to have a lower content of α-helix secondary structures and higher content of β-sheet than BF_4_ and H_2_PO_4_ anionic betaine ionic liquid modified lipases, further explaining the significant improvement in [BetaineC_16_][H_2_PO_4_]-CALB modified enzyme activity and stability.

**TABLE 3:** Secondary structure of enzyme and modified enzyme.

Enzyme	α-helix (%)	β-sheet (%)	β-turn (%)	Random coil (%)
CALB	46.8	11.2	26.9	16.9
[BetaineC_4_][Cl]-CALB	40.9	12.8	20.6	25.1
[BetaineC_8_][Cl]-CALB	37.9	15.3	22.4	24.4
[BetaineC_12_][Cl]-CALB	35.7	16.3	28.1	19.1
[BetaineC_16_][Cl]-CALB	31.5	25.3	20.3	22.6
[BetaineC_16_][BF_4_]-CALB	33.4	18.1	24.7	23.3
[BetaineC_16_][H_2_PO_4_]CALB	29.6	27.6	21.3	21.9

### 3.8 MALDI-TOF-MS Analysis

Peptide mass fingerprint analysis of CALB sites modified by ILs was performed using matrix-assisted laser desorption/ionization time-of-flight mass spectrometry (MALDI-TOF-MS). The results (shown in Supplementary Material) confirmed that CALB was modified with the successful identification of modification sites by comparison with the predicted peptide mass of native CALB.

### 3.9 Molecular Dynamics Simulation

#### 3.9.1 The Construction of Native and Modified CALB

The PDB file containing the optimal structure of non-natural amino acid residues obtained through field calculations of the online ATB server, was imported into Discovery Studio 2016 software (Koziara et al., 2014), with Lys124, Lys136, Lys290, and Lys308 sites selected for modification. Supplementary Figure S1 shows the CALB model before and after modification, using the examples of [BetaineC_4_][Cl]-CALB and [BetaineC_16_][Cl]-CALB.

#### 3.9.2 Changes in the Global Conformation of CALB Following Modification

**FIGURE 7:**
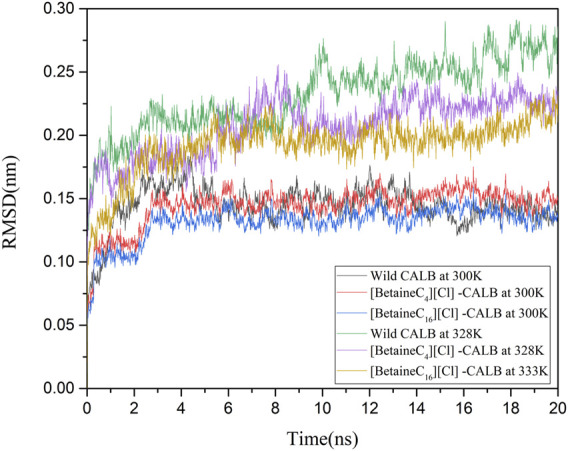
Changes in the RMSD values of native and modified enzymes at 300 K and their respective optimal temperatures.

The introduction of betaine ionic liquids caused different levels of change in the enzyme activity and enzymological properties of CALB. The changes in CALB were characterized using fluorescence and CD spectroscopy, as discussed, with modification using betaine ionic liquids causing structural changes and altering the catalytic performance of CALB. In order to comprehensively assess these enzymatic changes at a molecular level, molecular simulation was used, simulating the changes under different temperature conditions and RMSD values, with the simulation results shown in Figure 7. Comparing the native enzyme and the modified enzyme at a temperature of 300 K, the native enzyme fluctuated sharply within 0–6 ns and then reached an equilibrium state, while the modified enzyme reached an equilibrium state more rapidly, at about 3 ns, with the RMSD value of the modified enzyme being lower, indicating that the introduction of the betaine ionic liquid modifier made the structure of CALB more stable. At their respective optimal temperatures, the RMSD values of the native enzyme (328 K) and the modified enzymes were compared, showing that the RMSD value of the native enzyme fluctuated greatly at 328 K, indicating structural instability, which is consistent with the reported results of previous studies on the thermal stability of CALB. In contrast, after minor fluctuation within 0–4 ns, the modified enzyme RMSD still reached an equilibrium state rapidly. The RMSD value of [BetaineC_16_][Cl]-CALB at 333 K was higher than that of [BetaineC_4_][Cl]-CALB at 328 K. The occurrence of smaller RMSD values following modification with betaine ionic liquids with longer side chains, indicate more stable modified structures, which may be due to longer chain length modifiers having higher the molecular weights, which can reduce the structural vibration of enzyme protein molecules after heating.

#### 3.9.3 Energy Analysis

**FIGURE 8:**
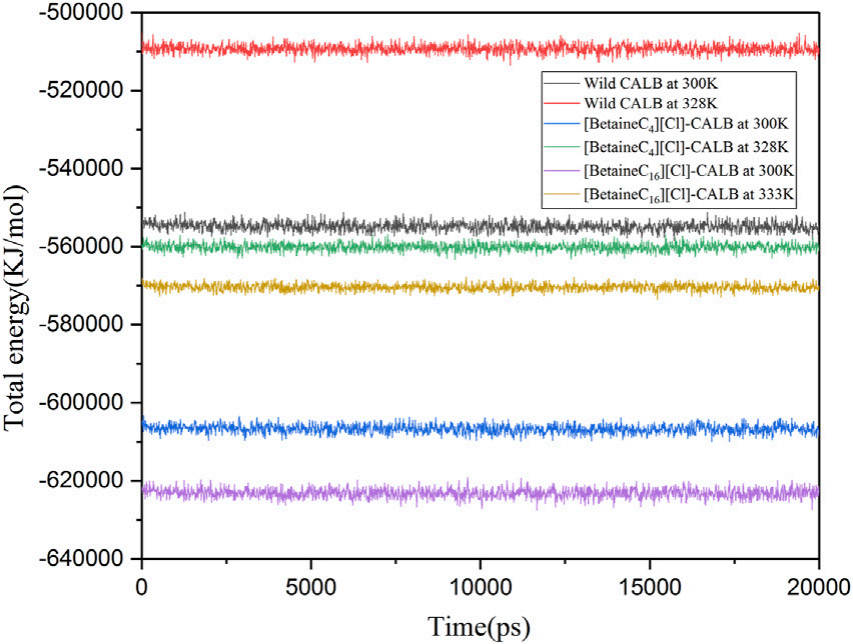
Changes in the total energy of native and modified enzymes at their respective optimal temperatures.

Wolynes and Dill et al. showed that protein molecules with lower energies have more stable molecular structures (Wolynes et al., 1995; Dill et al., 2008). Therefore, kinetic simulations were conducted for the native and modified enzymes at different temperatures for 20 ns. As shown in Figure 8, the total energy of [BetaineC_16_][Cl] modified CALB was lower than that of [BetaineC_4_][Cl] modified CALB at both room temperature and the enzyme specific optimal temperatures, indicating that longer modifier chain lengths allowed the enzyme protein to fold more easily, forming a more stable conformation.

#### 3.9.4 Flexibility of Amino Acid Residues

**FIGURE 9:**
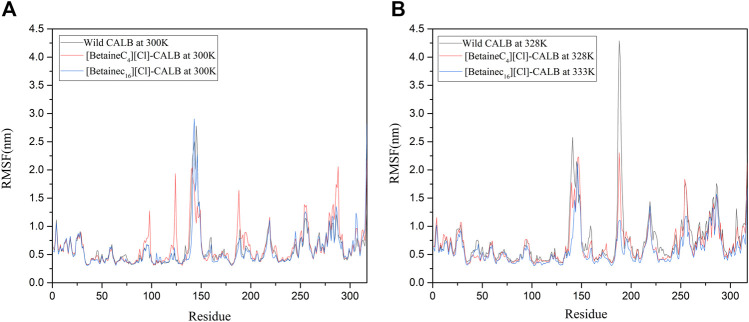
RMSF changes in the native and modified enzymes at their respective optimal temperatures, using the examples of [BetaineC_4_][Cl]-CALB and [BetaineC_16_][Cl]-CALB.

Changes in the local flexibility of amino acid residues in the enzyme protein has been reported to lead to changes in the catalytic activity and stability of lipases (Alcaraz et al., 2007; Fujiwara et al., 2009). As shown in Figure 9A, at 300 K, the difference in RMSF between the native and modified enzymes was not significant, with analysis showing that the ionic liquid modified CALB at the modification sites Lys124 and Lys290. The RMSF value in the region of Lys308 exhibited an upward trend, indicating that the introduction of a betaine ionic liquid only had a partial effect near the modification site, without damaging the CALB protein structure. As shown in Figure 9B, with increasing temperature, the RMSF value of the modified enzyme decreased compared with that of the native enzyme, indicating that the introduction of betaine ionic liquids could enhance the rigid structure of the enzyme protein. In addition, [BetaineC_16_][Cl] modified CALB was found to have significantly decreased RMSF values near 140, 180 and 225 at 333 K, indicating that betaine ionic liquids with longer side chains, induced more significant increases in the rigidity of the modified enzyme. This finding is consistent with the enzyme performance results of CD spectroscopic analysis, with longer betaine ionic liquid side chains resulting in a higher total content of β-sheet in the modified CALB, indicating that the enzyme protein has a stronger and more rigid structure.

#### 3.9.5 Analysis of Solvent Sccessibility Areas

**FIGURE 10:**
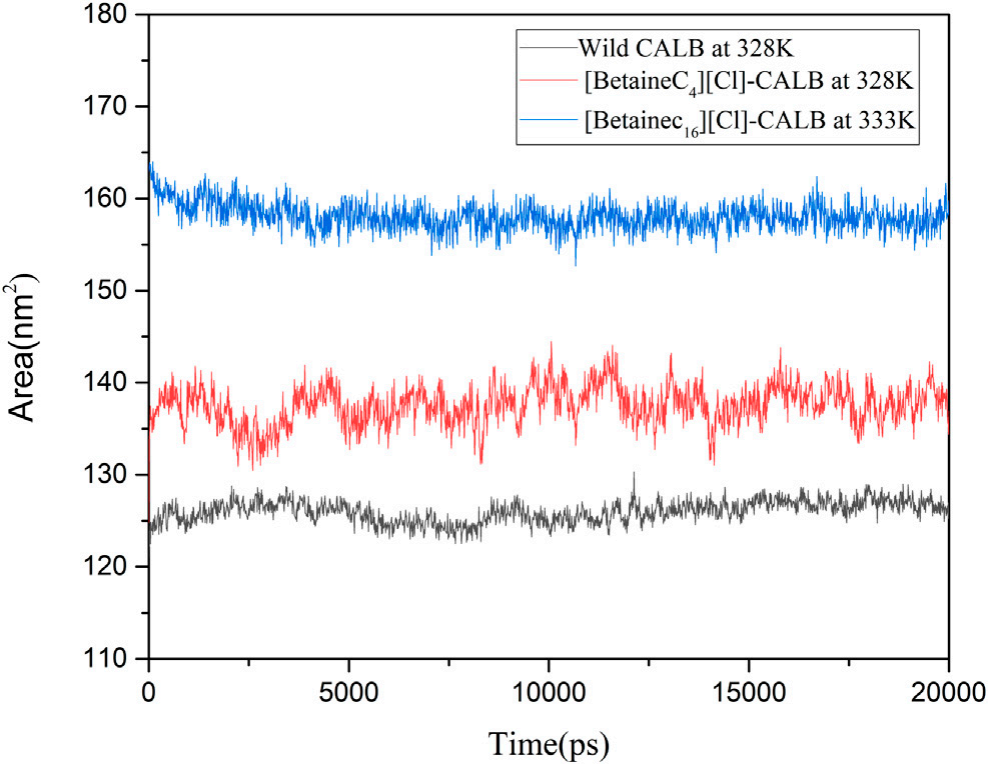
The hydrophobic and hydrophilic area of native and modified CALB using the examples of [BetaineC_4_][Cl]-CALB and [BetaineC_16_][Cl]-CALB.

Longo et al. modified the protease with a modifier containing a PEG group, resulting in the modified protease surface hydrophobic area being increased, which subsequently increased the thermal stability of the protease (Longo and Combes, 1997). In the present study, the SASA of the native and modified enzymes were calculated at their respective optimal temperatures, with the results shown in Figure 10. The SASA value of the modified enzymes were greater than that of the native enzyme, with the SASA value of [BetaineC_16_][Cl] modified CALB being the highest. Higher SASA values indicate a larger degree of exposure of amino acid residues of the enzyme protein. Longer hydrophobic alkane chains on the betaine ionic liquid can effectively prevent hydrophobic clusters which may cause instability when the enzyme protein contacts the surrounding water and therefore, the active conformation of the enzyme protein can be better protected at higher temperatures, improving the stability of the enzyme protein. In addition, an increase in the hydrophobic area may lead to a change in the hydrophobic environment near the enzyme binding pocket, enhancing the binding ability of the substrate and catalytic active center, further contributing to the increase in enzyme activity.

#### 3.9.6 The Radius of Rotation

The radius of rotation mainly reflects the volume and shape of the enzyme protein, with a larger radius of rotation resulting in larger enzyme protein expansion. As shown in Table 4, with an increase in temperature, the radius of rotation of the native enzyme becomes larger, indicating that the volume of the native enzyme expands to a certain extent, which may change the active conformation of the enzyme and thus reduce its activity. Minimal difference was observed in the radius of the native and modified enzymes, indicating that the introduction of betaine ionic liquids did not cause CALB volume expansion. In addition, with an increase in temperature, the rotation radius of modified enzymes did not change significantly, exhibiting good volume distribution and therefore, maintaining the conformation of the CALB enzyme protein at higher temperatures.

**TABLE 4:** Rg of native and modified CALB at different temperatures.

Temperature(K)	Rg (CALB)/nm	Rg ([BetaineC_4_][Cl]-CALB)/nm	Rg ([BetaineC_16_][Cl]-CALB)/nm
300	1.82	1.83	1.84
328	1.85	1.85	1.86
333	1.92	1.88	1.89

#### 3.9.7 Surface Charge Distribution

Gribenko et al. modified enzyme proteins using modifiers with different electrical properties, showing that the stability of the modified enzyme was improved compared with that of the native enzyme, indicating that the introduction of modifiers changes the charge distribution on the surface of enzyme proteins, affecting the catalytic performance of the enzyme (Gribenko et al., 2009). Mogharrab et al. showed that the introduction of a modifier could reduce the electrostatic interactions between residues near the horseradish peroxidase modification site and surrounding residues, improving the stability of the modified enzyme (Mogharrab et al., 2007). In the present study, as shown in Supplementary Figure S2, potential analysis was performed and modified CALB was found to have a higher potential distribution near the modified sites compared to the native enzyme. Therefore, the stability of the modified enzyme can be improved by neutralizing the charge of the protein in the presence of OH-, allowing the modified enzyme to maintain high activity under higher pH conditions.

#### 3.9.8 Protein–Water Radial Distribution Functions

**FIGURE 11:**
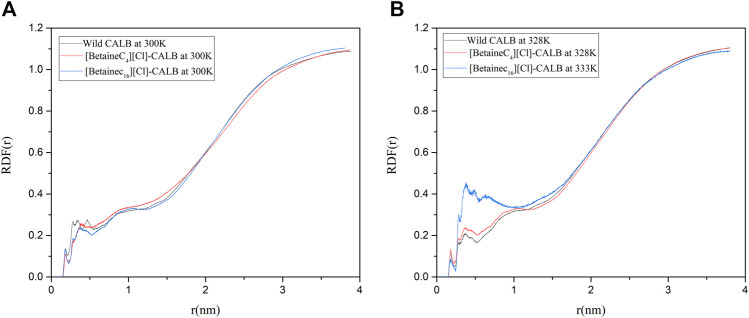
Radial distribution of water molecules around the active center of the native and modified enzymes at their respective optimal temperatures.

A water layer exists on the surface of enzyme proteins, resulting in water molecules being necessary to maintain the active conformation and catalytic performance of the enzyme. Liu et al. used PEG to modify LysB29 on insulin, enhancing the retention of water molecules during biocatalysis and increasing the interaction between water molecules and the surface of modified insulin, thereby increasing the thermal stability of the enzyme (Yang et al., 2011). Therefore, the introduction of modifiers can affect the distribution of essential water on the surface of protein molecules and the strength of interactions. In order to compare the changes in water molecules on the surface of the native and modified enzymes, the radial distribution function of water molecules were calculated. As shown in Figure 11, when the optimum temperature was reached, the distribution of water molecules near the active center of the modified enzyme significantly increased. In addition, the RDF value of [BetaineC_16_][Cl] modified CALB was significantly improved at 0–1 nm, indicating that the introduction of a modifier can not only improve the hydrophobicity of the enzyme surface, but also increases the hydrophilicity of the protein surface to some extent. An increase in the number of water molecules near the active center increases the number of essential water molecules participating in the enzymatic reaction, increasing the speed of the catalytic reaction and contributing to the increase in enzyme activity observed in the enzymatic performance experiment. In addition, the introduction of an ionic liquid increased the hydration layer density on the surface of the enzyme molecules, further stabilizing the active conformation of enzyme proteins, which is consistent with the previously observed increase in enzyme stability following modification.

## 4 Conclusion

In this study, betaine ionic liquids with different chain lengths and anions were designed and synthesized, then used for the chemical modification of CALB. The thermal stability of the enzyme activity of all modified enzymes were simultaneously improved to varying degrees, while longer chain length betaine ionic liquids resulted in better modification effects, as shown by the optimal performance of [BetaineC_16_][H_2_PO_4_] modified CALB. Analysis of the fluorescence spectra of the native and modified enzymes indicated that introduction of alkylated betaine ionic liquids resulted in a change in the microenvironment surrounding the fluorescent group of the enzyme protein. Longer alkyl chain length modifiers induced a more obvious decrease in fluorescence intensity, while the different modifier anions also caused a change in the fluorescence intensity of the modified enzyme. In addition, CD spectroscopy was used to identify the changes in the secondary structure of enzyme proteins, showing that with an increase in the modifier carbon chain length, the α-helix content exhibited a decreasing trend, while the β-sheet content showed an increasing trend. The decrease in α-helix content may be related to the increased enzyme activity, while the increased β-fold content may be due to the introduction of the ionic liquid, increasing the rigid structure on the surface of the enzyme protein, which is consistent with the change in modified enzyme properties such as thermal stability and organic solvent tolerance. Based on the enzymatic properties and spectroscopic structure characterization, molecular dynamics simulation was used to comprehensively investigate the mechanism of catalytic performance enhancement in modified enzymes. The results showed that the root mean square deviation (RMSD) and total energy of modified CALB were lower than those of native CALB, indicating that modification resulted in a more stable structure. The root mean square fluctuation (RMSF) calculations showed that the rigidity of the modified CALB was enhanced, while solvent accessibility area (SASA) calculations showed that both the hydrophilicity and hydrophobicity of the modified enzyme proteins were improved. The increase in radial distribution function (RDF) of water molecules confirmed that the number of water molecules surrounding the active sites also increased. Overall, it was observed that [BetaineC_16_][Cl] -CALB exhibited better simulation results than [BetaineC_4_][Cl]-CALB, further supporting the finding that modifiers with longer alkane chains have a more significant effect on enzyme activity and result in greater stability improvement. In summary, this study proposes an efficient method to improve the catalytic performance of lipases based on chemical modification with betaine ionic liquids, enriching the variety of ionic liquid modifiers available and broadening the application range of betaine ionic liquids in enzyme engineering. Furthermore, the mechanistic analysis of enzymatic activity in relation to the structural changes identified by molecular simulation and spectroscopy, provides fundamental knowledge for the rational design of chemically modified lipases, which can be successfully extended to the modification of other protein-enzymes.

## Data Availability

The original contributions presented in the study are included in the article/Supplementary Material, further inquiries can be directed to the corresponding authors.
