# Supplementary material for: Enhancing the Catalytic Performance of Candida antarctica Lipase B by Chemical Modification With Alkylated Betaine Ionic Liquids
Source: Front Bioeng Biotechnol. 2022 Feb 21;10:850890. doi: 10.3389/fbioe.2022.850890 (PMC8899502; doi:10.3389/fbioe.2022.850890)
Supplement: Supplementary file 1 [file DataSheet1.docx]

**Supporting information**


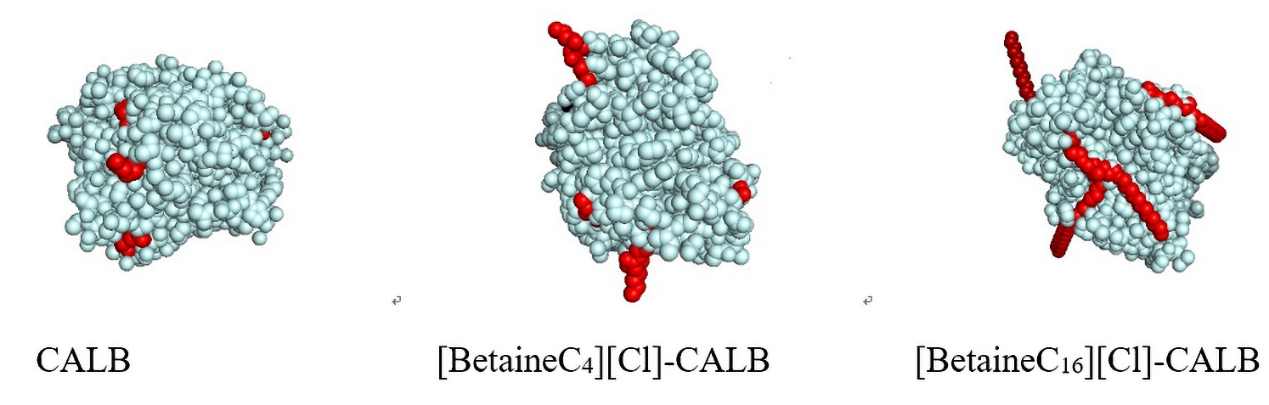


**FIGURE S1** | Schematic diagram of the structure of CALB before and after modification, using the examples of [BetaineC4][Cl]-CALB and [BetaineC16][Cl]-CALB


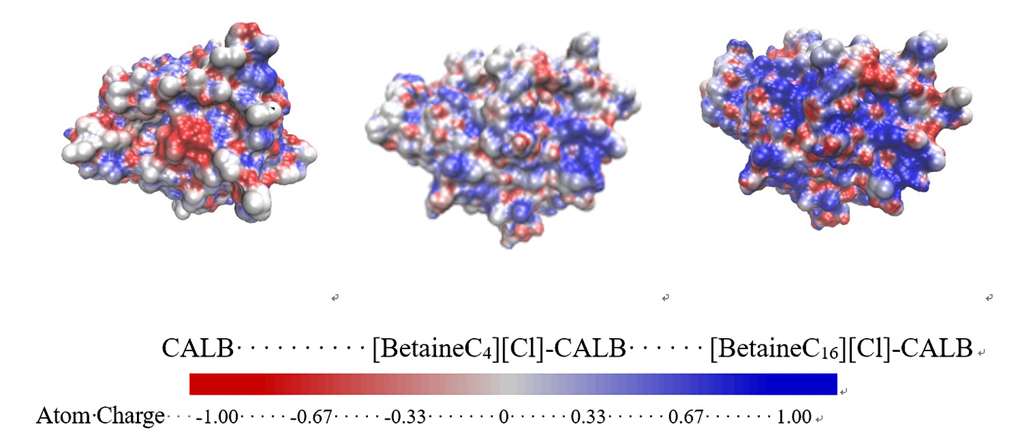


**FIGURE S2** | Surface charge distributions of the native and modified enzymes using the examples of [BetaineC4][Cl]-CALB and [BetaineC16][Cl]-CALB

（a）

[BetaineC_4_][Cl]，white solid，^1^H NMR (500 MHz，D_2_O) ，δ 0.7（t, *J*=10 Hz, 3H），δ 1.37（n, 2H），δ 1.7（n, 2H），δ 3.2（s, 6H），δ 3.49（n, 2H），δ 4.1（s, 2H）.

（b）

[BetaineC_8_][Cl]，white solid，^1^H NMR (500 MHz，D_2_O) ，δ 0.3（t, *J*=10 Hz, 3H），δ 1.24（n, 10H），δ 1.7（n, 2H），δ 3.19（s, 6H），δ 3.43（n, 2H），δ 3.9（s, 2H）.

（c）

[BetaineC_12_][Cl]，white solid，^1^H NMR(500 MHz，D_2_O) ，δ 0.81（t, *J*=10 Hz, 3H），δ 1.23（n, 18H），δ 1.67（n, 2H），δ 3.2（s, 6H），δ 3.57（n, 2H），δ 3.97（s, 2H）.

（d）

[BetaineC_16_][Cl]，white solid，^1^H NMR(500 MHz，D_2_O) ，δ 0.83（t, *J*=10 Hz, 3H），δ 1.23（n, 26H），δ 1.7（n, 2H），δ 3.2（s, 6H），δ 3.59（n, 2H），δ 3.83（s, 2H）.

Figure 1 Nuclear magnetic data of modifier

**MALDI-TOF-MS
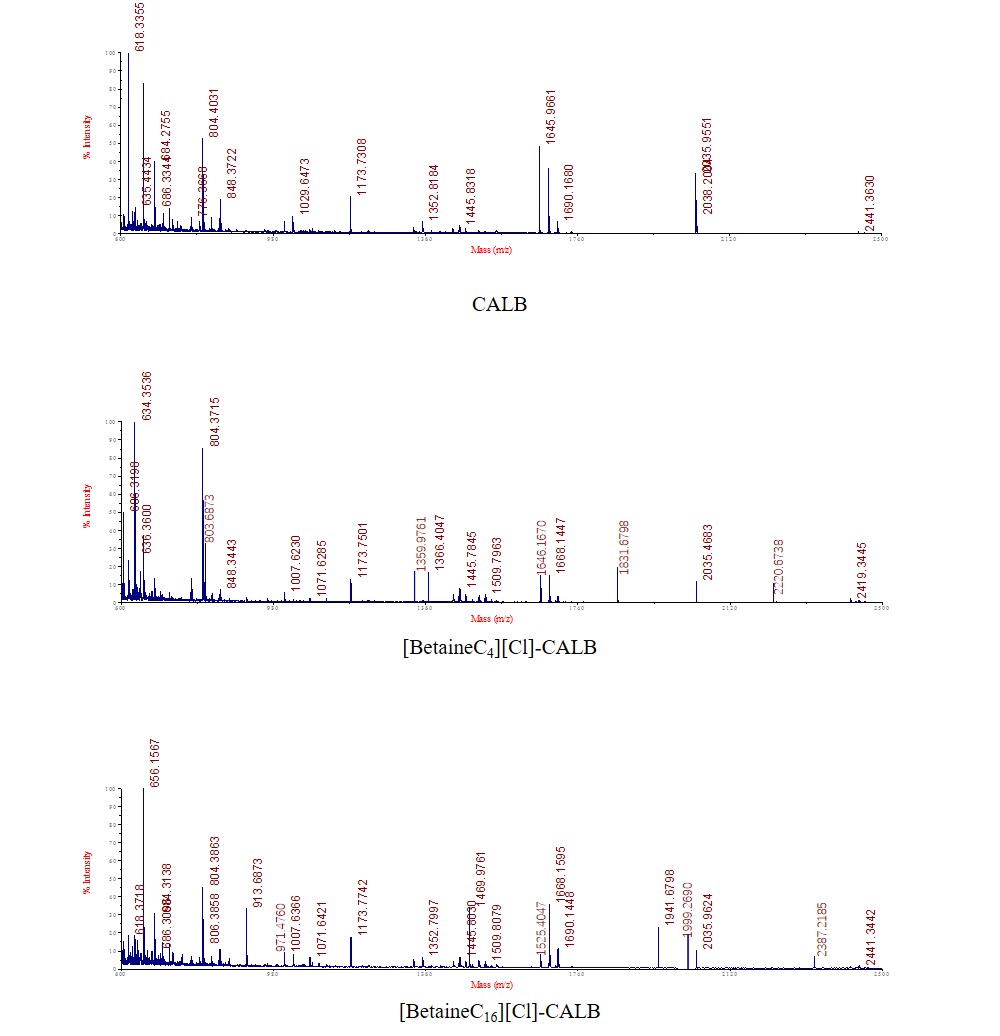
**

Figure 2 Peptide mass fingerprinting of native and modified CALBs

Table 1 shows the predicted and determined peptide mass fingerprint data. The peptide segments with significant changes in mass were identified and the sequences were analyzed, and then the modification sites were determined by analyzing the peptide mass fingerprint (Figure 2) and comparing with the mass of predicted peptide segments.

CALB modified by [BetaineC_4_][Cl] and [BetaineC_16_][Cl] had 4 modification sites Lys124, Lys136, Lys290 and Lys308.

Table 1 The analysis for peptide fragments from trypsin digest of CALB protein

| ILs | Modified sites | Expected mass | Measured mass | Δ mass | Target peptide sequences |
| --- | --- | --- | --- | --- | --- |
| C_4_-CALB-Cl | LYS124 | 618.3413 | 618.3355  803.6873 | 185.3518 | SKVDR |
|  | LYS136 | 1173.5230 | 1173.7742  1359.9761 | 186.2019 | LMAFAPDYK |
|  | LYS290 | 1645.9999 | 1646.1670  1831.6798 | 185.5128 | VAAAALLAPAAAAIVAGPK |
|  | LYS308 | 2035.9728 | 2035.9624  2220.6738 | 184.7114 | QNCEPDLMPYARPFAVGK |
| C_16_-CALB-Cl | LYS124 | 618.3413 | 618.3355  971.4760 | 353.1405 | SKVDR |
|  | LYS136 | 1173.5230 | 1173.7501  1525.4047 | 352.6546 | LMAFAPDYK |
|  | LYS290 | 1645.9999 | 1646.1550  1999.2690 | 353.1140 | VAAAALLAPAAAAIVAGPK |
|  | LYS308 | 2035.9728 | 2035.4683  2387.2185 | 352.7502 | QNCEPDLMPYARPFAVGK |

**The crystal structure of native and modified CALB**

It has confirmed that the crystal structure of CALB (PDB: 1TCA) which was downloaded from the PDB database, can be used as initial model, and the structure is stable and the reasonable calculation results can be obtained by molecular dynamics simulation. The protein was retained, and the redundant ligands and water molecules were deleted, the ionization state of amphoteric amino acids at pH 7.5 was selected.

The force field used for biological macromolecule is usually GROMACS-54a7, so the online calculation server Automated Topology Builder （ATB） and Repository （version 3.0） was used to calculate the force field of modified amino acid residue. The non-natural amino acid residue which was modified by[BetaineC_4_][Cl] and [BetaineC_16_][Cl], was shown in Figure.3. New force field parameters were obtained by calculation and added to the force field library. Specific operations were as follows: (1) The force field of non-natural amino acid residue needed to be recalculated by ATB software. (2) The force field parameters of non-natural amino acid which was calculated by ATB, were manually written into the information of amino acids residues in Gromacs software force field library (aminoacids.rtp). (3) Complete the information of hydrogen atom into the information of hydrogen bond in the force field file (aminoacids.hdb). (4) The name of the newly added non-natural amino acid residue was added to the residue library (residuetypes.dat). The partial information of non-natural amino acid residues is shown in Table 2.

(a)

(b)

Figure 3 The structure of KFR (a) and KST (b)

Table 2 Partial information of non-natural amino acids NLP in the top files

| [ KFR ] [ atoms ] | | | |
| --- | --- | --- | --- |
| atom | type | charge | cgnr |
| O | O | -0.595 | 1 |
| C | C | 0.098 | 1 |
| H02 | HC | 0.026 | 1 |
| CA | C | 0.281 | 1 |
| HA | HC | -0.058 | 1 |
| N | N | -0.955 | 1 |
| H | H | 0.263 | 1 |
| CB | C | -0.008 | 2 |
| CG | C | -0.027 | 2 |
| CD | C | 0.001 | 2 |
| CE | C | 0.293 | 2 |
| NZ | N | -0.530 | 2 |
| HZ | H | 0.354 | 2 |
| C9 | C | 0.326 | 3 |
| O11 | O | -0.468 | 3 |
| C8 | C | 0.189 | 4 |
| N1 | N | 0.327 | 4 |
| C2 | C | 0.153 | 4 |
| C3 | C | 0.138 | 4 |
| C4 | C | 0.111 | 4 |
| C5 | C | 0.033 | 4 |
| C6 | C | 0.037 | 4 |
| C7 | C | 0.011 | 4 |

| [ KFR ][ bonds ] | | | | |
| --- | --- | --- | --- | --- |
| ai | aj | funct | c0 | c1 |
| O | C | 2 | 0.1250 | 1.3400e+07 |
| C | H02 | 2 | 0.1110 | 4.8697e+06 |
| C | CA | 2 | 0.1490 | 1.4189e+07 |
| CA | HA | 2 | 0.1110 | 4.8697e+06 |
| CA | N | 2 | 0.1470 | 8.7100e+06 |
| CA | CB | 2 | 0.1530 | 7.1500e+06 |
| N | H | 2 | 0.1030 | 3.2991e+06 |
| CB | CG | 2 | 0.1530 | 7.1500e+06 |
| CG | CD | 2 | 0.1530 | 7.1500e+06 |
| CD | CE | 2 | 0.1530 | 7.1500e+06 |
| CE | NZ | 2 | 0.1470 | 8.7100e+06 |
| NZ | HZ | 2 | 0.1010 | 2.1076e+07 |
| NZ | C9 | 2 | 0.1340 | 1.0500e+07 |
| C9 | O11 | 2 | 0.1230 | 1.6600e+07 |
| C9 | C8 | 2 | 0.1540 | 4.2166e+06 |
| C8 | N1 | 2 | 0.1510 | 3.7279e+06 |
| N1 | C2 | 2 | 0.1510 | 3.7279e+06 |
| N1 | C3 | 2 | 0.1510 | 3.7279e+06 |
| N1 | C4 | 2 | 0.1540 | 4.2166e+06 |
| C4 | C5 | 2 | 0.1530 | 7.1500e+06 |
| C5 | C6 | 2 | 0.1540 | 4.2166e+06 |
| C6 | C7 | 2 | 0.1530 | 7.1500e+06 |

| [ KFR ][ angles ] | | | | | |
| --- | --- | --- | --- | --- | --- |
| ai | aj | ak | funct | angle | fc |
| 1 | C | H02 | 2 | 120.00 | 505.00 |
| 1 | C | CA | 2 | 126.00 | 640.00 |
| H02 | C | CA | 2 | 113.00 | 545.00 |
| C | CA | HA | 2 | 114.00 | 1559.41 |
| C | CA | N | 2 | 88.30 | 1201.92 |
| C | CA | CB | 2 | 111.00 | 530.00 |
| HA | CA | N | 2 | 120.00 | 505.00 |
| HA | CA | CB | 2 | 109.50 | 285.00 |
| N | CA | CB | 2 | 111.00 | 530.00 |
| CA | N | H | 2 | 109.50 | 425.00 |
| CA | CB | CG | 2 | 111.00 | 530.00 |
| CB | CG | CD | 2 | 111.00 | 530.00 |
| CG | CD | CE | 2 | 111.00 | 530.00 |
| CD | CE | NZ | 2 | 111.00 | 530.00 |
| CE | NZ | HZ | 2 | 116.00 | 465.00 |
| CE | NZ | C9 | 2 | 124.00 | 730.00 |
| HZ | NZ | C9 | 2 | 120.00 | 390.00 |
| NZ | C9 | O11 | 2 | 124.00 | 730.00 |
| NZ | C9 | C8 | 2 | 111.00 | 530.00 |
| O11 | C9 | C8 | 2 | 121.00 | 685.00 |
| C9 | C8 | N1 | 2 | 115.00 | 610.00 |
| C8 | N1 | C2 | 2 | 111.00 | 530.00 |
| C8 | N1 | C3 | 2 | 111.00 | 530.00 |
| C8 | N1 | C4 | 2 | 109.00 | 1680.51 |
| C2 | N1 | C3 | 2 | 109.00 | 1680.51 |
| C2 | N1 | C4 | 2 | 107.00 | 2726.16 |
| C3 | N1 | C4 | 2 | 110.00 | 285.00 |
| N1 | C4 | C5 | 2 | 115.00 | 610.00 |
| C4 | C5 | C6 | 2 | 109.50 | 520.00 |
| C5 | C6 | C7 | 2 | 111.00 | 530.00 |

| [ KST ][ atoms ] | | | | | |
| --- | --- | --- | --- | --- | --- |
| atom | type | charge | nr | res | chargegroup |
| N | N | -0.211000 | 1 | KST | 0 |
| CA | CT | -0.072000 | 2 | KST | 0 |
| C | CZ | 0.169000 | 3 | KST | 1 |
| O | O | -0.167000 | 4 | KST | 1 |
| CB | CT | -0.199000 | 5 | KST | 2 |
| CG | CT | -0.148000 | 6 | KST | 2 |
| CD | CT | -0.173000 | 7 | KST | 2 |
| CE | CT | -0.032000 | 8 | KST | 2 |
| NZ | N | -0.347000 | 9 | KST | 3 |
| C1 | C | 0.295000 | 10 | KST | 3 |
| C2 | CT | -0.156000 | 11 | KST | 3 |
| C3 | CT | -0.126000 | 12 | KST | 6 |
| C4 | CT | -0.181000 | 13 | KST | 6 |
| C5 | CT | -0.156000 | 14 | KST | 6 |
| C6 | CT | -0.162000 | 15 | KST | 6 |
| N1 | N3 | 0.046000 | 16 | KST | 5 |
| O1 | O | -0.373004 | 17 | KST | 3 |
| C7 | CT | -0.158000 | 18 | KST | 6 |
| C8 | CT | -0.159000 | 19 | KST | 6 |
| C9 | CT | -0.157000 | 20 | KST | 6 |
| C10 | CT | -0.158000 | 21 | KST | 6 |
| C11 | CT | -0.157000 | 22 | KST | 6 |
| C12 | CT | -0.158000 | 23 | KST | 6 |
| C13 | CT | -0.157000 | 24 | KST | 6 |
| C14 | CT | -0.158000 | 25 | KST | 6 |
| C15 | CT | -0.158000 | 26 | KST | 6 |
| C16 | CT | -0.158000 | 27 | KST | 6 |
| C17 | CT | -0.158000 | 28 | KST | 6 |
| C18 | CT | -0.211000 | 29 | KST | 6 |
| HT | H | 0.140000 | 30 | KST | 0 |
| HA | H1 | 0.141000 | 31 | KST | 0 |
| HB2 | HC | 0.108000 | 32 | KST | 2 |
| HB1 | HC | 0.108000 | 33 | KST | 2 |
| HG2 | HC | 0.086000 | 34 | KST | 2 |
| HG1 | HC | 0.086000 | 35 | KST | 2 |
| HD1 | HC | 0.104500 | 36 | KST | 2 |
| HD2 | HC | 0.104500 | 37 | KST | 2 |
| HE2 | H1 | 0.080500 | 38 | KST | 2 |
| HE1 | H1 | 0.080500 | 39 | KST | 2 |
| HZ | H | 0.268000 | 40 | KST | 3 |
| C19 | CT | -0.196500 | 41 | KST | 5 |
| C20 | CT | -0.196500 | 42 | KST | 5 |
| H32 | HP | 0.133000 | 43 | KST | 6 |
| H31 | HP | 0.133000 | 44 | KST | 6 |
| H22 | HP | 0.153500 | 45 | KST | 3 |
| H21 | HP | 0.153500 | 46 | KST | 3 |
| H42 | HC | 0.107500 | 47 | KST | 6 |
| H41 | HC | 0.107500 | 48 | KST | 6 |
| H52 | HC | 0.091000 | 49 | KST | 6 |
| H51 | HC | 0.091000 | 50 | KST | 6 |
| H62 | HC | 0.096000 | 51 | KST | 6 |
| H61 | HC | 0.096000 | 52 | KST | 6 |
| H72 | HC | 0.075500 | 53 | KST | 6 |
| H71 | HC | 0.075500 | 54 | KST | 6 |
| H81 | HC | 0.086000 | 55 | KST | 6 |
| H82 | HC | 0.086000 | 56 | KST | 6 |
| H91 | HC | 0.078500 | 57 | KST | 6 |
| H92 | HC | 0.078500 | 58 | KST | 6 |
| H102 | HC | 0.082500 | 59 | KST | 6 |
| H101 | HC | 0.082500 | 60 | KST | 6 |
| H112 | HC | 0.079000 | 61 | KST | 6 |
| H111 | HC | 0.079000 | 62 | KST | 6 |
| H121 | HC | 0.081000 | 63 | KST | 6 |
| H122 | HC | 0.081000 | 64 | KST | 6 |
| H131 | HC | 0.079000 | 65 | KST | 6 |
| H132 | HC | 0.079000 | 66 | KST | 6 |
| H141 | HC | 0.079500 | 67 | KST | 6 |
| H142 | HC | 0.079500 | 68 | KST | 6 |
| H152 | HC | 0.078500 | 69 | KST | 6 |
| H151 | HC | 0.078500 | 70 | KST | 6 |
| H162 | HC | 0.079500 | 71 | KST | 6 |
| H161 | HC | 0.079500 | 72 | KST | 6 |
| H172 | HC | 0.078000 | 73 | KST | 6 |
| H171 | HC | 0.078000 | 74 | KST | 6 |
| H182 | HC | 0.073667 | 75 | KST | 6 |
| H183 | HC | 0.073667 | 76 | KST | 6 |
| H181 | HC | 0.073667 | 77 | KST | 6 |
| HH12 | HP | 0.148167 | 78 | KST | 5 |
| HH22 | HP | 0.148167 | 79 | KST | 5 |
| HH32 | HP | 0.148167 | 80 | KST | 5 |
| HH11 | HP | 0.148167 | 81 | KST | 5 |
| HH21 | HP | 0.148167 | 82 | KST | 5 |
| HH31 | HP | 0.148167 | 83 | KST | 5 |

| [ KST ][ angles ] | | | | |
| --- | --- | --- | --- | --- |
| ai | aj | ak | theta | cth |
| N | CA | C | 1.1238e+02 | 5.6317e+02 |
| N | CA | CB | 1.1161e+02 | 5.5145e+02 |
| N | CA | HA | 1.0888e+02 | 4.1673e+02 |
| CA | N | HT | 1.1768e+02 | 3.8325e+02 |
| CA | C | O | 1.8000e+02 | 4.8476e+02 |
| CA | CB | CG | 1.1151e+02 | 5.2635e+02 |
| CA | CB | HB2 | 1.0980e+02 | 3.8744e+02 |
| CA | CB | HB1 | 1.0980e+02 | 3.8744e+02 |
| C | CA | CB | 1.1171e+02 | 5.3723e+02 |
| C | CA | HA | 1.0924e+02 | 4.0501e+02 |
| CB | CA | HA | 1.0956e+02 | 3.8828e+02 |
| CB | CG | CD | 1.1151e+02 | 5.2635e+02 |
| CB | CG | HG2 | 1.0980e+02 | 3.8744e+02 |
| CB | CG | HG1 | 1.0980e+02 | 3.8744e+02 |
| CG | CB | HB2 | 1.0980e+02 | 3.8744e+02 |
| CG | CB | HB1 | 1.0980e+02 | 3.8744e+02 |
| CG | CD | CE | 1.1151e+02 | 5.2635e+02 |
| CG | CD | HD1 | 1.0980e+02 | 3.8744e+02 |
| CG | CD | HD2 | 1.0980e+02 | 3.8744e+02 |
| CD | CG | HG2 | 1.0980e+02 | 3.8744e+02 |
| CD | CG | HG1 | 1.0980e+02 | 3.8744e+02 |
| CD | CE | NZ | 1.1161e+02 | 5.5145e+02 |
| CD | CE | HE2 | 1.0956e+02 | 3.8828e+02 |
| CD | CE | HE1 | 1.0956e+02 | 3.8828e+02 |
| CE | CD | HD1 | 1.0980e+02 | 3.8744e+02 |
| CE | CD | HD2 | 1.0980e+02 | 3.8744e+02 |
| CE | NZ | C1 | 1.2069e+02 | 5.3053e+02 |
| CE | NZ | HZ | 1.1768e+02 | 3.8325e+02 |
| NZ | CE | HE2 | 1.0888e+02 | 4.1673e+02 |
| NZ | CE | HE1 | 1.0888e+02 | 4.1673e+02 |
| NZ | C1 | C2 | 1.1518e+02 | 5.5898e+02 |
| NZ | C1 | O1 | 1.2305e+02 | 6.2091e+02 |
| C1 | NZ | HZ | 1.1755e+02 | 4.0417e+02 |
| C1 | C2 | N1 | 1.1073e+02 | 5.4810e+02 |
| C1 | C2 | H22 | 1.0877e+02 | 3.9246e+02 |
| C1 | C2 | H21 | 1.0877e+02 | 3.9246e+02 |
| C2 | C1 | O1 | 1.2320e+02 | 5.6400e+02 |
| C2 | N1 | C3 | 1.0966e+02 | 5.2384e+02 |
| C2 | N1 | C19 | 1.0966e+02 | 5.2384e+02 |
| C2 | N1 | C20 | 1.0966e+02 | 5.2384e+02 |
| C3 | C4 | C5 | 1.1151e+02 | 5.2635e+02 |
| C3 | C4 | H42 | 1.0980e+02 | 3.8744e+02 |
| C3 | C4 | H41 | 1.0980e+02 | 3.8744e+02 |
| C3 | N1 | C19 | 1.0966e+02 | 5.2384e+02 |
| C3 | N1 | C20 | 1.0966e+02 | 5.2384e+02 |
| C4 | C3 | N1 | 1.1421e+02 | 5.3723e+02 |
| C4 | C3 | H32 | 1.0980e+02 | 3.8744e+02 |
| C4 | C3 | H31 | 1.0980e+02 | 3.8744e+02 |
| C4 | C5 | C6 | 1.1151e+02 | 5.2635e+02 |
| C4 | C5 | H52 | 1.0980e+02 | 3.8744e+02 |
| C4 | C5 | H51 | 1.0980e+02 | 3.8744e+02 |
| C5 | C4 | H42 | 1.0980e+02 | 3.8744e+02 |
| C5 | C4 | H41 | 1.0980e+02 | 3.8744e+02 |
| C5 | C6 | C7 | 1.1151e+02 | 5.2635e+02 |
| C5 | C6 | H62 | 1.0980e+02 | 3.8744e+02 |
| C5 | C6 | H61 | 1.0980e+02 | 3.8744e+02 |
| C6 | C5 | H52 | 1.0980e+02 | 3.8744e+02 |
| C6 | C5 | H51 | 1.0980e+02 | 3.8744e+02 |
| C6 | C7 | C8 | 1.1151e+02 | 5.2635e+02 |
| C6 | C7 | H72 | 1.0980e+02 | 3.8744e+02 |
| C6 | C7 | H71 | 1.0980e+02 | 3.8744e+02 |
| N1 | C2 | H22 | 1.0790e+02 | 4.0668e+02 |
| N1 | C2 | H21 | 1.0790e+02 | 4.0668e+02 |
| N1 | C3 | H32 | 1.0790e+02 | 4.0668e+02 |
| N1 | C3 | H31 | 1.0790e+02 | 4.0668e+02 |
| N1 | C19 | HH11 | 1.0790e+02 | 4.0668e+02 |
| N1 | C19 | HH21 | 1.0790e+02 | 4.0668e+02 |
| N1 | C19 | HH31 | 1.0790e+02 | 4.0668e+02 |
| N1 | C20 | HH12 | 1.0790e+02 | 4.0668e+02 |
| N1 | C20 | HH22 | 1.0790e+02 | 4.0668e+02 |
| N1 | C20 | HH32 | 1.0790e+02 | 4.0668e+02 |
| C7 | C6 | H62 | 1.0980e+02 | 3.8744e+02 |
| C7 | C6 | H61 | 1.0980e+02 | 3.8744e+02 |
| C7 | C8 | C9 | 1.1151e+02 | 5.2635e+02 |
| C7 | C8 | H81 | 1.0980e+02 | 3.8744e+02 |
| C7 | C8 | H82 | 1.0980e+02 | 3.8744e+02 |
| C8 | C7 | H72 | 1.0980e+02 | 3.8744e+02 |
| C8 | C7 | H71 | 1.0980e+02 | 3.8744e+02 |
| C8 | C9 | C10 | 1.1151e+02 | 5.2635e+02 |
| C8 | C9 | H91 | 1.0980e+02 | 3.8744e+02 |
| C8 | C9 | H92 | 1.0980e+02 | 3.8744e+02 |
| C9 | C8 | H81 | 1.0980e+02 | 3.8744e+02 |
| C9 | C8 | H82 | 1.0980e+02 | 3.8744e+02 |
| C9 | C10 | C11 | 1.1151e+02 | 5.2635e+02 |
| C9 | C10 | H102 | 1.0980e+02 | 3.8744e+02 |
| C9 | C10 | H101 | 1.0980e+02 | 3.8744e+02 |
| C10 | C9 | H91 | 1.0980e+02 | 3.8744e+02 |
| C10 | C9 | H92 | 1.0980e+02 | 3.8744e+02 |
| C10 | C11 | C12 | 1.1151e+02 | 5.2635e+02 |
| C10 | C11 | H112 | 1.0980e+02 | 3.8744e+02 |
| C10 | C11 | H111 | 1.0980e+02 | 3.8744e+02 |
| C11 | C10 | H102 | 1.0980e+02 | 3.8744e+02 |
| C11 | C10 | H101 | 1.0980e+02 | 3.8744e+02 |
| C11 | C12 | C13 | 1.1151e+02 | 5.2635e+02 |
| C11 | C12 | H121 | 1.0980e+02 | 3.8744e+02 |
| C11 | C12 | H122 | 1.0980e+02 | 3.8744e+02 |
| C12 | C11 | H112 | 1.0980e+02 | 3.8744e+02 |
| C12 | C11 | H111 | 1.0980e+02 | 3.8744e+02 |
| C12 | C13 | C14 | 1.1151e+02 | 5.2635e+02 |
| C12 | C13 | H131 | 1.0980e+02 | 3.8744e+02 |
| C12 | C13 | H132 | 1.0980e+02 | 3.8744e+02 |
| C13 | C12 | H121 | 1.0980e+02 | 3.8744e+02 |
| C13 | C12 | H122 | 1.0980e+02 | 3.8744e+02 |
| C13 | C14 | C15 | 1.1151e+02 | 5.2635e+02 |
| C13 | C14 | H141 | 1.0980e+02 | 3.8744e+02 |
| C13 | C14 | H142 | 1.0980e+02 | 3.8744e+02 |
| C14 | C13 | H131 | 1.0980e+02 | 3.8744e+02 |
| C14 | C13 | H132 | 1.0980e+02 | 3.8744e+02 |
| C14 | C15 | C16 | 1.1151e+02 | 5.2635e+02 |
| C14 | C15 | H152 | 1.0980e+02 | 3.8744e+02 |
| C14 | C15 | H151 | 1.0980e+02 | 3.8744e+02 |
| C15 | C14 | H141 | 1.0980e+02 | 3.8744e+02 |
| C15 | C14 | H142 | 1.0980e+02 | 3.8744e+02 |
| C15 | C16 | C17 | 1.1151e+02 | 5.2635e+02 |
| C15 | C16 | H162 | 1.0980e+02 | 3.8744e+02 |
| C15 | C16 | H161 | 1.0980e+02 | 3.8744e+02 |
| C16 | C15 | H152 | 1.0980e+02 | 3.8744e+02 |
| C16 | C15 | H151 | 1.0980e+02 | 3.8744e+02 |
| C16 | C17 | C18 | 1.1151e+02 | 5.2635e+02 |
| C16 | C17 | H172 | 1.0980e+02 | 3.8744e+02 |
| C16 | C17 | H171 | 1.0980e+02 | 3.8744e+02 |
| C17 | C16 | H162 | 1.0980e+02 | 3.8744e+02 |
| C17 | C16 | H161 | 1.0980e+02 | 3.8744e+02 |
| C17 | C18 | H182 | 1.0980e+02 | 3.8744e+02 |
| C17 | C18 | H183 | 1.0980e+02 | 3.8744e+02 |
| C17 | C18 | H181 | 1.0980e+02 | 3.8744e+02 |
| C18 | C17 | H172 | 1.0980e+02 | 3.8744e+02 |
| C18 | C17 | H171 | 1.0980e+02 | 3.8744e+02 |
| HB2 | CB | HB1 | 1.0758e+02 | 3.2970e+02 |
| HG2 | CG | HG1 | 1.0758e+02 | 3.2970e+02 |
| HD1 | CD | HD2 | 1.0758e+02 | 3.2970e+02 |
| HE2 | CE | HE1 | 1.0846e+02 | 3.2803e+02 |
| C19 | N1 | C20 | 1.0966e+02 | 5.2384e+02 |
| H32 | C3 | H31 | 1.0758e+02 | 3.2970e+02 |
| H22 | C2 | H21 | 1.0758e+02 | 3.2970e+02 |
| H42 | C4 | H41 | 1.0758e+02 | 3.2970e+02 |
| H52 | C5 | H51 | 1.0758e+02 | 3.2970e+02 |
| H62 | C6 | H61 | 1.0758e+02 | 3.2970e+02 |
| H72 | C7 | H71 | 1.0758e+02 | 3.2970e+02 |
| H81 | C8 | H82 | 1.0758e+02 | 3.2970e+02 |
| H91 | C9 | H92 | 1.0758e+02 | 3.2970e+02 |
| H102 | C10 | H101 | 1.0758e+02 | 3.2970e+02 |
| H112 | C11 | H111 | 1.0758e+02 | 3.2970e+02 |
| H121 | C12 | H122 | 1.0758e+02 | 3.2970e+02 |
| H131 | C13 | H132 | 1.0758e+02 | 3.2970e+02 |
| H141 | C14 | H142 | 1.0758e+02 | 3.2970e+02 |
| H152 | C15 | H151 | 1.0758e+02 | 3.2970e+02 |
| H162 | C16 | H161 | 1.0758e+02 | 3.2970e+02 |
| H172 | C17 | H171 | 1.0758e+02 | 3.2970e+02 |
| H182 | C18 | H183 | 1.0758e+02 | 3.2970e+02 |
| H182 | C18 | H181 | 1.0758e+02 | 3.2970e+02 |
| H183 | C18 | H181 | 1.0758e+02 | 3.2970e+02 |
| HH12 | C20 | HH22 | 1.0758e+02 | 3.2970e+02 |
| HH12 | C20 | HH32 | 1.0758e+02 | 3.2970e+02 |
| HH22 | C20 | HH32 | 1.0758e+02 | 3.2970e+02 |
| HH11 | C19 | HH21 | 1.0758e+02 | 3.2970e+02 |
| HH11 | C19 | HH31 | 1.0758e+02 | 3.2970e+02 |
| HH21 | C19 | HH31 | 1.0758e+02 | 3.2970e+02 |
